# Retrospective Analysis of Wood Anatomical Traits Reveals a Recent Extension in Tree Cambial Activity in Two High-Elevation Conifers

**DOI:** 10.3389/fpls.2017.00737

**Published:** 2017-05-08

**Authors:** Marco Carrer, Daniele Castagneri, Angela L. Prendin, Giai Petit, Georg von Arx

**Affiliations:** ^1^Dipartimento Territorio e Sistemi Agro-Forestali, Universitá degli Studi di PadovaLegnaro, Italy; ^2^Swiss Federal Institute for Forest, Snow and Landscape Research WSLBirmensdorf, Switzerland; ^3^Climatic Change and Climate Impacts, Institute for Environmental SciencesGeneva, Switzerland

**Keywords:** cambial activity, *Larix decidua* Mill., *Picea abies* (L.) Karst., treeline, tree-ring anatomy, xylem phenology

## Abstract

The study of xylogenesis or wood formation is a powerful, yet labor intensive monitoring approach to investigate intra-annual tree growth responses to environmental factors. However, it seldom covers more than a few growing seasons, so is in contrast to the much longer lifespan of woody plants and the time scale of many environmental processes. Here we applied a novel retrospective approach to test the long-term (1926–2012) consistency in the timing of onset and ending of cambial activity, and in the maximum cambial cell division rate in two conifer species, European larch and Norway spruce at high-elevation in the Alps. We correlated daily temperature with time series of cell number and lumen area partitioned into intra-annual sectors. For both species, we found a good correspondence (1–10 days offset) between the periods when anatomical traits had significant correlations with temperature in recent decades (1969–2012) and available xylogenesis data (1996–2005), previously collected at the same site. Yet, results for the 1926–1968 period indicate a later onset and earlier ending of the cambial activity by 6–30 days. Conversely, the peak in the correlation between annual cell number and temperature, which should correspond to the peak in secondary growth rate, was quite stable over time, with just a minor advance of 4–5 days in the recent decades. Our analyses on time series of wood anatomical traits proved useful to infer on past long-term changes in xylogenetic phases. Combined with intensive continuous monitoring, our approach will improve the understanding of tree responses to climate variability in both the short- and long-term context.

## Introduction

Trees respond throughout their life to short- and long-term environmental influences by tuning their growth rate, and this leaves permanent imprints in the woody tissue. They respond to a wealth of physical (e.g., climate, geomorphological processes, etc.) (Hughes et al., 2010; Stoffel et al., 2010) and biological drivers (e.g., population dynamics, biotic disturbances, etc.) (Swetnam et al., 1985; Black and Abrams, 2003). Trees are thus considered among the most valuable natural archives of past environmental conditions (Swetnam et al., 1999; Bradley, 2013) and a unique source for annual variability in forest biomass and carbon allocation (Bouriaud et al., 2005; Babst et al., 2014). Moreover, there has been increasing interest in analyzing (intra-annual) tree-ring characteristics to better understand, for example, tree growth responses to environmental variability and extreme events (Battipaglia et al., 2014) and performance of different species provenances (Schreiber et al., 2015). Recent efforts based on intra-annual tree-ring characteristics aim at deciphering the effect of climate on the whole xylogenetic process, i.e., the sequence of phases that, starting from the environmental input, through photosynthetic and cambial activities, results in the synthesis of new woody tissue (Fukuda, 1996; Rossi et al., 2012; Cuny et al., 2014).

Most xylogenetic investigations follow two major lines of research: one analyzing the biochemical processes connected with the biosynthesis of wood components (e.g., cellulose and lignin) (Boerjan et al., 2003), the role of specific genes (Hertzberg et al., 2001) and hormonal signaling (Aloni, 1995). In the other, analyses are at tissue scale to investigate general aspects of wood formation dynamics related mainly to cambium phenology and the role of environmental factors (Camarero et al., 2010; Rossi et al., 2013). Both these approaches are highly labor intensive in terms of sampling, sample preparation and subsequent analyses, as they entail directly monitoring the processes over time. This significantly limits the time frame of investigations, which is seldom longer than a few growing seasons, with most studies confined to a single year (Rossi et al., 2013). This limitation is not a serious concern for biochemical-related analyses because time is usually not the key dimension in this research field. However, it might be relevant in studies focusing on timing and rate of the phenological phases of xylem cells production (e.g., cell division, enlargement and maturation through cell wall thickening and plasmolysis of cell content), and on climate influence on them (Rossi et al., 2007; Camarero et al., 2010; Gričar et al., 2014). Extrapolating the inferences obtained from data collected over one to a few growing seasons to a long-term context could lead to biased interpretations (Rossi et al., 2011; Boulouf Lugo et al., 2012). For example, any scenario on future wood production, carbon sequestration or species phenology in a warming world could result in significant uncertainties if the model forecasts were based on a few growing seasons in recent, and usually warmer, years.

Dendroanatomy, i.e., the analysis of xylem-cell features along dated tree-ring series, might offer a longer-term perspective on wood formation processes (Fonti et al., 2010). Recent advances in sample processing and image analysis allow time frames as long as the more established ring-width or wood-density studies to be covered, together with detailed measurements of multiple traits on hundreds to thousands of cells for each annual growth ring (von Arx and Carrer, 2014; Prendin et al., 2017). Since both the dendroanatomical and xylogenetic approaches operate at cellular level, relating dendroanatomy to key environmental factors might also allow inferences on cambial activity to be extended from a few years to decades. However, up to now, only a few empirical studies have explored long-term effects of climate variability on the corresponding year-to-year change in wood anatomical traits and the related consequences for conifer tree physiology and growth (Olano et al., 2012; Fonti et al., 2013; Pacheco et al., 2016; Pellizzari et al., 2016). Furthermore, most of these investigations usually adopted a classical dendroclimatological approach to define the associations between climate and wood-anatomical parameters (e.g., using monthly resolved weather records and averaging the anatomical properties of the whole rings), therefore remaining disconnected from the cambium dynamics studies in terms of temporal resolution. As a result, the xylogenesis and dendroanatomical approaches have often targeted the same objective of quantifying the association between climate and tree-ring growth, but always at two different time scales and resolutions.

In this study, by coupling long-term daily temperature records with detailed wood anatomical analyses, we aimed to integrate the high-resolution and mechanistic, but short-term, xylogenetic approach with the longer-term, but less detailed, tree-ring analyses. The intention was to extract information about xylem dynamics, such as the onset and ending of cambial activity and the peak rate of cambial cell division, through a retrospective analysis of intra-annual anatomical features (tracheid-lumen area and tracheid number) along multi-decadal tree-ring series. To this end we collected samples from *Larix decidua* Mill. (larch) and *Picea abies* (L.) Karst. (spruce) in a timberline site at high elevation in the Alps where xylogenetic analyses had already been conducted in both species and temperature revealed as being the key factor for cambial activity (Rossi et al., 2007, 2008). We then compared the previous results on cambial dynamics with our findings obtained through dendroanatomical analyses.

## Materials and Methods

### Study Site and Climate

The study area is located on a North-East facing slope at high elevation above the Cortina d’Ampezzo basin in the Eastern Italian Alps (46°30′N, 12°07′E, 2100 m a.s.l.) where several investigations involving dendroecology and intensive tree-growth monitoring have been underway for more than 15 years (Anfodillo et al., 1998; Castagneri et al., 2015). The sample site is a typical mixed timberline forest with European larch (*Larix decidua* Mill., hereafter larch), Swiss stone pine (*Pinus cembra* L.), and Norway spruce (*Picea abies* Karst., hereafter spruce), with irregular spatial distribution of trees and low canopy density. Soils are shallow and calcareous. Mean annual precipitation is 1080 mm, with a maximum in June (125 mm), and usually falls as snow from November to early May. Mean annual temperature is 6.7°C with daily extremes ranging from -25 to +30°C. Meteorological data came from the Cortina d’Ampezzo station (1230 m a.s.l), located less than 4 km from the sample site. This station provides the longest (1926–2012) daily record in the region and, despite the lower elevation, is fully representative of the day-to-day temperature variability in the area (see Supplementary Figure S1).

### Sampling and Cell Measurement

Two 5-mm cores per tree were collected at breast height on the cross-slope sides of the trunk from 12 trees per species. We followed the classical dendroecological protocol (Schweingruber et al., 1990), selecting healthy dominant or co-dominant mature trees with no visible scars or signs of recent injuries in an attempt to reduce as much as possible any effects of non-climatic external influences such as small-scale disturbances (Supplementary Table S1).

Ring widths were measured to the nearest 0.01 mm using a measuring table. To match each growth ring with the year of its formation we crossdated all the series (Stokes and Smiley, 1968). The accuracy of this operation was verified using the COFECHA crossdating software (Holmes, 1983). For anatomical analysis we selected the best of the two cores per tree. This selection was based on plain and complete tree-ring sequences, avoiding rotten parts, callus tissues, reaction wood or mechanical damage. Cores from seven spruce and six larch trees met these quality criteria.

**FIGURE 1:**
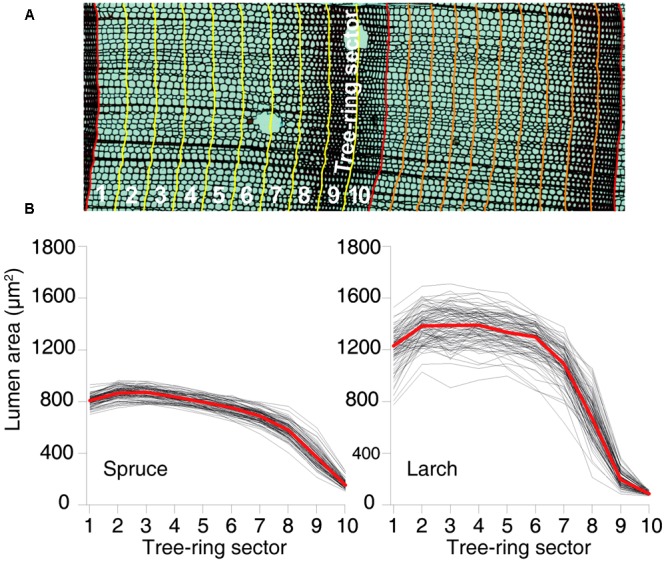
**Tree-ring partitioning and profiles of cell-lumen area within the rings. (A)** Graphical representation of tree-ring partitioning using 10 sectors. **(B)** Dimension of the mean lumen area by sector for each year between 1926 and 2012 for both species. Red lines represent the overall mean values.

Xylem anatomical analyses followed the protocol proposed by von Arx et al. (2016). In brief, cores were split in 4–5 cm long pieces and transversal sections (15–20 μm thick) were prepared with a rotary microtome (Leica, Heidelberg, Germany), stained with safranin (1% in distilled water) and permanently fixed with Eukitt (BiOptica, Milan, Italy). Multiple overlapping digital images covering the entire samples were captured with a light microscope at 40× magnification (Nikon Eclipse 80) and stitched together with PTGui (New House Internet Service B.V., The Netherlands). Tracheid cell lumina in the stitched images (**Figure 1A**) were then quantified using the specialized image-analysis tool ROXAS (von Arx and Carrer, 2014).

We considered two anatomical parameters: (1) mean cell-lumen area (MCA), which is related to the duration of and turgor during cell enlargement (Taiz and Zeiger, 2010; Cuny et al., 2014) and (2) cell number (CN), a parameter directly linked to the rate and duration of cambial activity (Vaganov et al., 2006). However, rather than applying the classical approach of selecting and measuring a few radial cell files along each ring (Fritts et al., 1991; Panyushkina et al., 2003; Bryukhanova and Fonti, 2013; Fonti et al., 2013) and considering the findings by Seo et al. (2014) that increasing the number of cell files improves the stability and reliability of time series, we opted for a more thorough and unbiased scheme measuring all the cells within each image. This corresponds to an average of 40–60 cell files per ring. In total, 3873 rings and more than 6,000,000 cells were processed (1818 rings and 2105 ± 1578 cells per ring (CN) for spruce; 2062 and 1068 ± 991 for larch).

### Tree-Ring and Cell Chronologies

Besides the conventional tree-ring width (TRW) chronology, i.e., dated annual time series averaged at species level, we built 11 additional anatomical chronologies for each species: 1 with the number of CN and 10 based on the cell-lumen area. These 10 chronologies were built by splitting each ring into 10 tangential bands or sectors of equal width from early- to latewood, assigning each tracheid cell to the corresponding sector within the respective ring based on available positional information, and calculating the MCA (**Figure 1A**) for each of the 10 intra-ring sectors. Chronologies were then formed using MCA of the first, second, etc., sector of each ring. While the resulting chronologies ranged from earlywood to latewood, they did not assume any subjective definition of these two intra-ring parts, and represented consecutive, although partially overlapping, time windows during the phases of tree-ring formation within the growing season. Dividing the tree rings in 10 sectors was considered a reasonable compromise between robust sample size (number of cells per sector) and sufficient intra-annual disaggregation of the tree-ring data (temporal resolution).

Tree-ring width and CN series were first standardized to remove the typical age-size related trends due to adjoining new rings to an increasing stem girth. This was accomplished by a spline detrending with a 50% frequency cutoff response at 30 years that preserves inter-annual to decadal variability in the resulting standard chronologies (Cook et al., 1990). Series of cell-lumen area also usually exhibit a long-term trend particularly visible in the first ring sectors, where most of the larger cells typically devoted to hydraulic transport occur. This increasing trend is strictly connected to ontogenetic height growth and is mostly evident during the first decades of tree life when height growth rate is maximized; it then levels off once the tree reaches its maximum height, at around 150/200 years in this area (Carrer and Urbinati, 2004; Carrer et al., 2015). Given the age of the sampled trees, always older than 180 years (mean, min, max age was 230, 184, 345 years for spruce and 362, 259, 487 years for larch, cf. Supplementary Table S1), the last 87 years (1926–2012), which corresponded to the time span for which climate data was available, includes no evident long-term ontogenetic trend. Standardization was therefore not considered necessary.

Several descriptive statistics were computed to assess key properties of each chronology: the mean value and standard deviation (SD), which estimates the variability of measurements for the whole series; the mean sensitivity (MS), which is an indicator of the mean relative change between consecutive years and is calculated as the absolute difference between the values of successive years divided by their mean. MS represents a measure of the year-to-year growth variability and is adopted together with SD to assess the high-frequency variation in the series (Fritts, 1976); the mean correlation between series from different trees (Rbar); and the expressed population signal (EPS), which quantifies the degree to which a chronology matches a hypothetical population chronology and estimates the level of year-by-year parameter variability shared by trees at the same site. Higher values of Rbar and EPS indicate greater similarity in the annual patterns among trees and a better representation of overall stand behavior by the mean chronologies (Wigley et al., 1984). To assess the common patterns of variability among the chronologies of anatomical parameters we used principal component analysis (PCA) (Jolliffe, 2002) computed on the correlation matrix for the period 1926–2012, which is the period adopted to test the correlation with temperature. The significance of the principal components was verified with a randomization test and applying the Rnd-Lambda stopping rule (Peres-Neto et al., 2005). Scatter plots of the weighting coefficients for the first two PCs were used to display the similarities among variables.

### Defining the Relationships between Cell Parameters and Temperature

Cambial activity at this elevation occurs within a fairly short growing season that usually extends from May to September. Previous investigations highlighted that temperature is the most significant driver for xylogenesis in the site (Rossi et al., 2007, 2008). In order to match past cambium phenological phases and cell division rate with meteorological conditions, we computed the temperature-growth correlations between wood anatomical chronologies and daily temperature records from 1926 to 2012. To better cope with the short-term temperature influence on cell parameters and to go beyond the rough and arbitrary aggregation into 12 months (Fonti et al., 2013), we opted for a 15-day time frame, which is similar to those usually applied in cambium dynamics analyses. Daily temperature data were therefore averaged over a 15-day moving window shifted at daily step, and running Pearson correlations with the TRW, CN and cell-sector chronologies were then computed between April 1st and October 30th of the ring formation year. This time span included a buffer of about 1 month before the beginning and after the end of the typical growing season for both species in the area (Rossi et al., 2008). The underlying idea was that the permanent imprint on xylem cells induced by the temperature variability during the course of cell formation could be analyzed through temperature-cell size correlations computed within the same period. Indeed, no significant correlation should be observed outside the phases of cambial activity because short-term temperature variability cannot leave a sign in completely formed (and dead) xylem cells. On the contrary, at the onset (ending) of cambial activity, cells start (finish) being directly influenced by temperature, so a corresponding onset (ending) of the sequential series of significant correlations should be observed with the 15-day moving temperature window. To retrospectively define the peak rate in cambial cell division we considered the number of cells, CN. Since this variable is directly related to the cambial activity, we assumed that the peak of cell production would match the corresponding peak in temperature sensitivity.

To assess the consistency of the temperature-growth correlations over time we split the 87-year period into two 43-year sub-periods (1926–1968 and 1969–2012). In addition, to compare the species behavior under cold and warm conditions, the latter representative of the likely future climate scenario (Barriopedro et al., 2011; IPCC, 2013), we selected the 30 warmest and 30 coldest years of the entire record for the April to September period, which differed by 2.1°C (see Supplementary Table S3 and Figure S2). This period corresponds to the growing season but includes 1 month before the onset to account for possible lagged responses. For both 30-year periods we calculated the temperature-growth correlations as described for the whole study period. Given the number of tests involved (Peres-Neto et al., 2005), the significance of temperature/growth relationships was tested accounting for multiplicity and adopting the False Discovery Rate approach (Benjamini and Hochberg, 1995).

## Results

### Chronology Features

The chronologies’ descriptive statistics (**Table 1**) demonstrated that our tree-ring partitioning approach allows reliable cell-lumen time series to be built, i.e., series with a common and persisting year-to-year variability among trees. All chronologies showed significant differences (Mann–Whitney *U* test, Supplementary Tables S1, S2) between species, with spruce usually having more but smaller CN than larch (**Table 1** and **Figure 1B**). Similar to ring width, the year-to-year variability expressed by MS was always higher in larch than in spruce. However, for the MCA, both species showed higher year-to-year variability (MS) in the last sectors with a peak in the 9th. The strength of the common signal between series of the same species and the quality of the chronologies, as assessed through the Rbar and EPS statistics, pointed out that anatomical parameters had lower performances than TRW. This general tendency was not maintained in spruce by the chronologies of the 9th and 10th sectors where Rbar and EPS were higher than the corresponding TRW values, reaching the remarkable level of 0.557 and 0.910, respectively.

**Table 1:** Descriptive statistics for ring-width, cell-number and mean cell-lumen area (MCA) of the sector chronologies in the two species.

	Chronology	Mean ± SD	MS	Rbar	EPS
	**Spruce**				
	Ring width	0.899 @ 0.233	0.163	0.448	0.867
	Cell number	1637 @ 454	0.168	0.296	0.727
Mean lumen area (MCA)	1st sector	712 @ 91	0.099	0.133	0.551
	2nd sector	772 @ 97	0.097	0.153	0.591
	3rd sector	774 @ 95	0.094	0.144	0.574
	4th sector	745 @ 90	0.092	0.146	0.578
	5th sector	712 @ 87	0.093	0.127	0.539
	6th sector	670 @ 87	0.102	0.163	0.610
	7th sector	614 @ 90	0.124	0.183	0.641
	8th sector	512 @ 98	0.179	0.275	0.752
	9th sector	309 @ 89	0.291	0.463	0.863
	10th sector	121 @ 30	0.234	0.557	0.910
	
	**Larch**				
	Ring width	0.757 @ 0.401	0.307	0.557	0.883
	Cell number	659 @ 217	0.250	0.430	0.819
Mean lumen area (MCA)	1st sector	1204 @ 225	0.193	0.211	0.616
	2nd sector	1377 @ 212	0.151	0.213	0.619
	3rd sector	1387 @ 230	0.167	0.306	0.726
	4th sector	1379 @ 204	0.145	0.348	0.762
	5th sector	1344 @ 200	0.141	0.268	0.688
	6th sector	1270 @ 201	0.158	0.347	0.762
	7th sector	1096 @ 215	0.213	0.262	0.681
	8th sector	698 @ 241	0.409	0.298	0.718
	9th sector	236 @ 105	0.400	0.280	0.660
	10th sector	95 @ 31	0.219	0.174	0.558

*Chronology statistics represent the mean value and standard deviation expressed in different units (*mm* for ring width and *μm*^*2*^ for MCA), mean sensitivity (MS), mean interseries correlation (Rbar), and the expressed population signal (EPS). All statistics are computed for the 1926–2012 common period (the same interval used for correlations with climate).*

**FIGURE 2:**
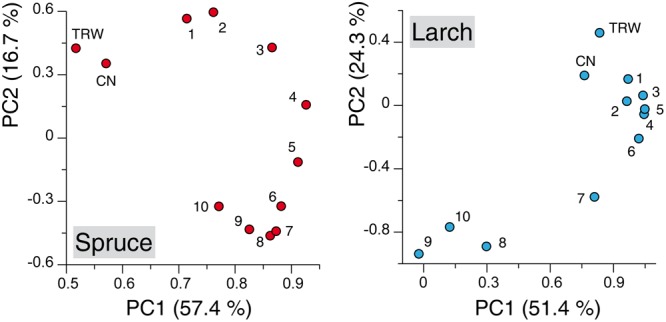
**Principal component analysis (PCA) applied to the chronologies of wood anatomical parameters.** Scatter plots of weighting coefficients for PC1 and PC2 calculated on chronologies built with tree-ring width (TRW), cell number (CN) and with mean cell-lumen area (MCA) for each of the 10 tree-ring sectors. Numbers in the scatter plots refer to sectors ranging from earlywood (1, 2, 3,...) to latewood (..., 8, 9, 10) (See **Figure 1** for comparison). Axis labels report the percentage of variance expressed by each component.

Principal component analysis performed with the TRW and anatomical chronologies resulted for both species in two significant components: together they explained 74.1 and 75.8% of the variance for spruce and larch, respectively. The ordination along the two axis revealed several patterns (**Figure 2**): (i) a clear separation along the first axis between TRW, CN, and MCA chronologies in spruce while in larch the first two variables highlighted a much more similar mode to the earlywood sectors; (ii) a smooth and subtle transition between the first-sector chronologies and the last ones in spruce, where a separation among most of the tree-ring sectors was possible based mainly on the PC2 loadings, and (iii) a much clearer partition, along both components, of the first- versus last-sector chronologies in larch with the 7th-sector chronology in an intermediate position.

### Long-Term Cambium Dynamics and Their Relationships with Temperature

**FIGURE 3:**
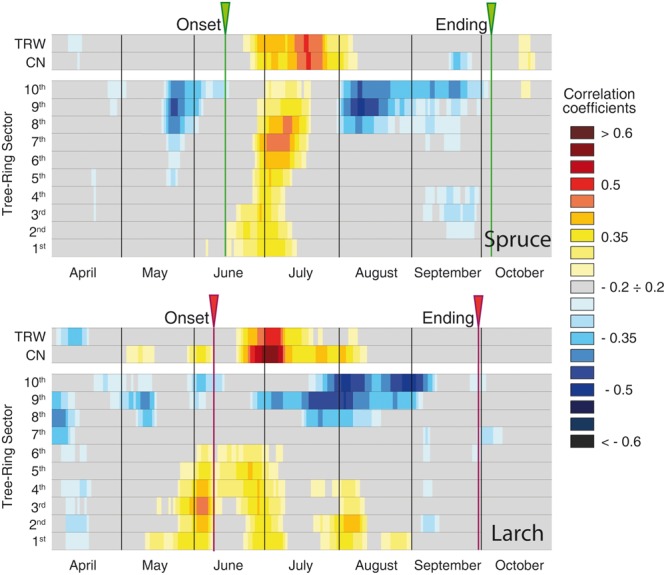
**Temperature-growth correlations (1926–2012), for the two species, computed between anatomical parameters (cell number and sectors chronologies) and ring-width chronologies, and mean temperature expressed as a 15-day moving window.** Correlation coefficients are represented for each time window shifted at daily steps and are coded according to the color scale on the right. Gray cases are not significant, colored boxes are significant (*p* ≤ 0.05) with the standard approach (i.e., considering each test as independent). The arrows at the top of each plot correspond to the onset and ending dates detected by intensive monitoring with dendrometers or following xylem phenology (Rossi et al., 2006, 2008).

**FIGURE 4:**
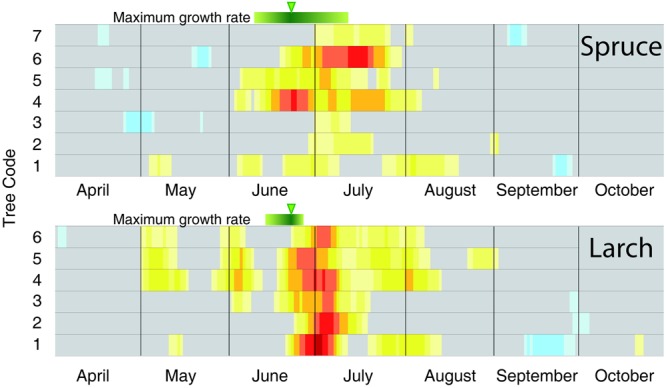
**Individual-tree temperature-growth correlations for the two species.** Correlations (1926–2012) have been computed at tree level between cell-number time series and mean temperature expressed as a 15-day moving window. The representation, color coding and significance of the correlation coefficients are the same as in **Figure 3**. The green bars and arrows at the top of each plot correspond to the maximum growth rate detected by intensive monitoring with dendrometers and xylem phenology (Rossi et al., 2006, 2008).

The global overview of temperature-growth correlations for all the chronologies showed both similarities and differences between the two species. In both species there was an evident contrast between the responses of the last sectors, being negatively correlated with temperature, and the first ones with mainly positive correlations. The periods when these significant correlations with temperature occurred matched the intensive monitoring data well (i.e., dendrometers continuous monitoring or following xylem phenology) (**Figures 3, 4** and **Table 2**). CN featured a correlation profile similar to TRW that culminated around the first days of July for larch and slightly later for spruce, highlighting a temporal offset between the species. The first sectors began to respond sensitively to temperature variability between mid-May and mid-June until mid-July. Significant correlations emerged earlier for larch than spruce and thus confirmed a temporal offset. The last sectors (generally from the 8th to the 10th in both species) seemed sensitive to temperature until mid- to late September. The only notable difference (cf. also **Table 2** in the 1969–2012 period) was observed in larch at the beginning of the growing season when intensive monitoring identified the onset of cambial activity around June 8th, whereas a significant correlation between the first sector and temperature was already detected around May 12th. Almost all parameters evidenced: (i) an offset between the species, with spruce showing a delay compared to larch in the onset and ending of its sensitivity to temperature; (ii) a sequential start of the significant positive correlation with temperature from the first to the subsequent sectors, mainly in the first sectors but also in the last ones in spruce; (iii) a period of significant negative temperature sensitivity in the last sectors mainly in late May/early June in spruce and in a few spots between April and early June in larch, which was far ahead of the development of latewood cells; and (iv) a strong negative correlation in the last three sectors for the second part of the growing season.

**Table 2:** Day of the year (DOY) when the different phenological phases were recorded or detected adopting two different approaches: intensive monitoring (with dendrometers continuous monitoring or following xylem phenology) and dendroanatomy.

	Period	Max growth rate	Onset	Ending	*Approach*
**Spruce**	1926–1968	187	172	268	*Dendroanatomy*
	1969–2012	183	168	276	*Dendroanatomy*
	1996–2005	173	166	277	*Intensive monitoring*
**Larch**	1926–1968	187	143	250	*Dendroanatomy*
	1969–2012	182	119	280	*Dendroanatomy*
	1996–2005	173	159	272	*Intensive monitoring*

**FIGURE 5:**
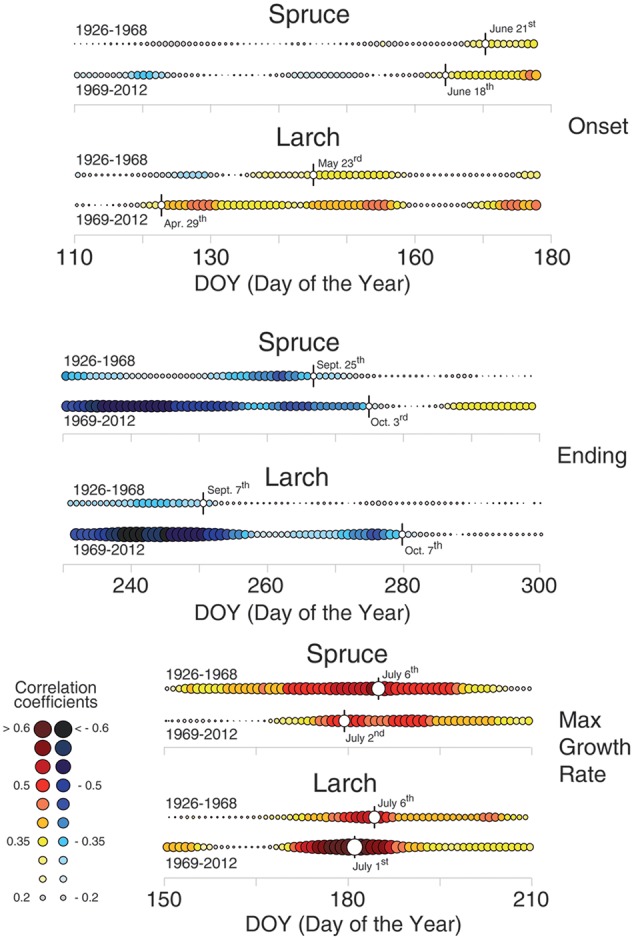
**Temperature-growth correlations for the two species split in two sub-periods (1926–1968 and 1969–2012).** Temperature-growth correlations computed between mean temperature expressed as a 15-day moving window and cell-lumen chronologies of the first (growth onset) and last (growth end) sector, and cell number (maximum growth rate). Correlation coefficients are computed and the color coding assigned as in **Figures 3, 4**. White circles with vertical flag represent the first (for the 1st sector; onset) or last (10th sector; ending) day of continuous significant correlations, and the peak of correlation values for cell number/maximum growth rate, respectively. Significant (*p* < 0.05) correlation values are higher than |0.30| .

*Data of intensive monitoring taken from Rossi et al. (2006, 2008). For dendroanatomy the DOY corresponds to the first (onset) or last day (ending of the growing season) of a continuous series of at least 10 significant correlations computed between temperature and the MCA chronology of the 1st or 10th sector. For maximum growth rate the DOY coincides with the day with the highest significant correlation values. See **Figure 5** for further details.*

To better compare the peak in the CN correlation with the corresponding information from intensive monitoring, we performed the same analysis at individual tree level (**Figure 4**). Here the maximum sensitivity to temperature in CN tended to converge around the end of June, with larch being more coherent than spruce. In accordance with previous results, these dates (**Figure 4**) also matched well, although with a minor delay of ca. 1 week, with the maximum rate of cambial activity recorded in many conifers in temperate and boreal zones (Rossi et al., 2006).

Dividing the investigation period in two sub-periods (1926–1968 and 1969–2012) and computing the temperature-growth correlations for the first and last sector (**Figure 5**), we observed a general shift in recent decades toward earlier correlations for the first sectors, up to the end of April (Day of Year – DOY 119 in larch) and a corresponding delay for the last ones toward the end of the growing season (early October, DOY 280). Between the two periods larch revealed larger shifts than spruce whereas both species responded similarly in CN, showing just a minor advance in the peak of the correlation with temperature in recent decades.

**FIGURE 6:**
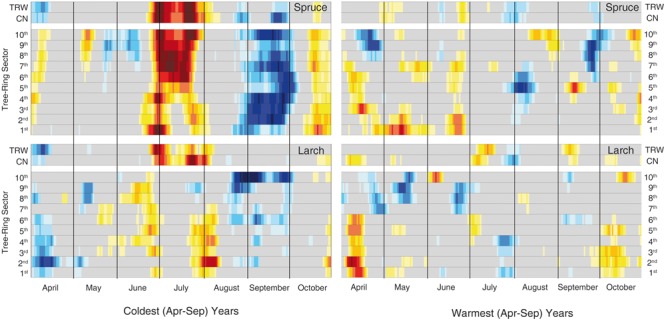
**Temperature-growth correlations for the two species in the extreme years.** Correlations have been computed between the same anatomical, tree-ring and temperature parameters as in **Figure 3** but selecting just the 30 coldest and warmest years (based on mean April-September temperature) within the period 1926–2012. The representation and color coding are the same as **Figure 3**, however, in this case the threshold for significant (*p* < 0.05) correlations is set at |0.35| according to the fewer years considered than in the previous analysis.

Finally, the analyses of the 30 coldest and 30 warmest years (April to September) (**Figure 6**, Supplementary Table S4 and Figure S2) showed a general reduction of the significant correlations in the warmest years for both species, while a clear increase in the temperature sensitivity of spruce emerged in the cold years compared to the whole 87-year period.

## Discussion

### Quality and Reliability of the Anatomical Chronologies

The statistics used to assess chronology quality of anatomical variables are in line with those of TRW, with most of them being lower but a few, especially in spruce, even higher. This shows that, by measuring all cells instead of only the cells along a few radial files it is possible to create reliable anatomical chronologies even for intra-ring sectors. This is a key finding. Indeed, up to now poor performances of most previous cell anatomical time-series questioned the potential application of dendroanatomical parameters in the reconstruction of past environmental conditions or tree functioning (Olano et al., 2012, 2013; Seo et al., 2012). However, previous investigations have shown that inter-tree correlation (Rbar) or chronology common signal (EPS) can be decoupled from the climatic responses (Yasue et al., 2000; Fonti and Garcia-Gonzalez, 2004; Fonti et al., 2013; Olano et al., 2013). That is, weak quality metrics of anatomical chronologies might turn in a robust climatic signal as already proved for various species and anatomical traits (Yasue et al., 2000; Campelo et al., 2010; Olano et al., 2013). In our case, quality statistics of anatomical chronologies were comparably robust (e.g., with an EPS value higher than the commonly accepted threshold of 0.85), which likely demonstrates the high level of accuracy and representativeness of our measurement approach. The other descriptive statistics confirmed the different anatomical and behavioral traits between the two species: the larger tracheids in larch, which allow a more efficient water flow, are consistent with its usually higher water spending strategy compared to spruce (Anfodillo et al., 1998). The higher TRW year-to-year variability in larch, expressed by MS, is characteristic for this deciduous conifer compared to the evergreen spruce that only replaces part of its foliage each year, thus reducing inter-annual variability (Carrer et al., 1998). The increasing trend in MS from the first to the last sectors in both species is likely due to the strong hydraulic constraints mainly present in the larger cells of the first sectors whose main function is water transport. In the latewood this function is much reduced or even absent, which may make these tracheids more susceptible to recording other environmental inputs such as climate (temperature in our case) variability. This explanation is in line with the observation that maximum latewood density is usually the best proxy in tree-ring related climate reconstruction in temperature-limited ecosystems (Frank et al., 2007; Büntgen et al., 2015).

### Characteristics of Lumen-Area Sectors and Correspondence to Period of Cambial Activity

Various wood anatomical parameters can provide distinct environmental information, as already observed in both broadleaves (Perez-de-Lis et al., 2016; Castagneri et al., 2017b) and conifers (Olano et al., 2012; Bryukhanova and Fonti, 2013). However, together with the well-known similarities between CN and TRW, the observed separation between these two variables and the sector chronologies in PCA, especially for spruce, demonstrates that different signals can also be detected within the same parameter (lumen area in this case) when considering the relative position of each tracheid within the tree ring. Tracheids from the first to the last ring sector correspond to successive time windows within the growing season. This is evident in **Figure 3**, with the smooth sequential propagation of the positive cell-lumen correlation with temperature across the sectors, and with the further postponed negative correlations for the three latewood sectors. In addition, the first and last dates of these significant correlations correspond well to the onset and ending dates provided by intensive monitoring in the same area with the only mismatch being the onset phase in larch (**Table 2**) (Rossi et al., 2008). In this deciduous species cambium reactivation seems not to be directly triggered by a rise in temperature, as in evergreen conifers, but rather by an increase in auxin concentration in the cambial region synthesized after budbreak in young expanding leaves (Oribe and Kubo, 1997; Begum et al., 2013). Leaf phenology surveys conducted in the same area between 1997 and 2000 (Anfodillo, unpublished data) support this hypothesis, reporting that leaf unfolding occurred in larch around the first days in May (DOY 120), the same period identified in our retrospective analysis for the last decades (**Table 2**).

### Linking Temperature Responses of Cell Size to Underlying Xylogenetic Processes

The opposite temperature correlations among the early- and latewood sectors chronologies represent the two different facets of the positive effect of temperature on wood formation at this high-elevation site. Here, xylogenesis benefits from warm temperature in two different ways depending on the timing of cell production: (i) during the first part of the season, when earlywood tracheids are produced, the positive effect is visible mainly in cell-lumen dimension, i.e., during the enlargement phase. In this case, when water is not limiting, warm temperatures would firstly favor cambial activity, leading to the formation of a wider cambial zone and therefore the production of a higher number of larger cells throughout the season (Kirdyanov et al., 2003; Vaganov et al., 2006) and secondly, changes in the physical properties of cell walls such as flexibility and hydraulic conductivity that further promote the formation of larger conduits (Antonova and Stasova, 1997); (ii) in the second part of the season negative correlations with temperature are likely not directly associated with a detrimental effect on lumen area (MCA) but rather to a positive stimulus on cell wall thickening. Warmer temperature would increase the centripetal deposition of wall material per cell and also its kinetics, with the direct effect of increasing cell wall thickness but with the side cost of reduced lumen area (Cuny et al., 2014). In spruce, with generally smaller tracheids, this negative effect on MCA of latewood sectors seems particularly strong and outweighs the benefits of temperature occurring just a few days before (the positive correlations in early July). Cuny et al. (2014) showed that, in the absence of major environmental stresses, the decrease in cell size along the ring is the major driver for the parallel change in cell wall thickness, while the amount of wall material per cell remains fairly constant throughout the season. According to those authors, up to two-thirds of the cell wall thickness is likely due to the corresponding change in lumen area. Nonetheless, the highly significant correlations with temperature for the latewood sectors suggest that the remaining third of variability still permits a significant climatic imprint. The correspondence between these negative temperature correlations and cell wall thickness dynamics is also confirmed by the significant synchrony with the intensive cambium phenology data (**Table 2**), where the ending of cambial activity closely matches the last significant correlations detected in the latewood sectors for the last decades with dendroanatomy (Rossi et al., 2008).

### Cell Number Is a Better Indicator of Maximum Growth Rate than Ring Width

Several investigations noted the strong relationships between CN and TRW in conifers (Vaganov et al., 2006; Olano et al., 2012), and our results support this robust association in both species. However, compared to TRW, CN shows a stronger correlation with temperature and a closer temporal correspondence with the maximum cambium growth rates detected with intensive monitoring, particularly in larch. Rossi et al. (2006) showed that conifers in cold temperate and boreal regions synchronize maximum growth rate around the time of maximum day length and not during the warmest period of the year. This could imply a partial decoupling from weather conditions at least in some xylogenetic processes, with photoperiod pacing the annual growth rate and temperature mainly affecting metabolic activities during cell production and differentiation. Our results provide further insight into these patterns: (i) the importance of photoperiod for cambium phenology emerged even on a longer time scale and seemed stable over time for both species (Körner and Basler, 2010) and (ii) the growth rate is in any case significantly sensitive to temperature and this sensitivity peaks in the period of maximum growth rate.

### Distinct Signals from Lumen-Area Sectors Depending on General Temperature Regime

The importance of temperature for wood cell formation at our high-elevation site is further stressed when considering the warmest and coldest April-September seasons. In the warmest years we observed a clear degradation of the temperature signal that contrasts with the strong responses in the coldest years, especially for spruce. This might suggest a relaxation of the limiting conditions experienced by trees at high elevation, in some cases detected as a degradation of the climatic signal in the TRW in recent decades (Briffa et al., 1998; D’Arrigo et al., 2008). Given that 15 out of the last 20 years, i.e., the period in which most xylogenetic studies have been conducted, are among the 30 warmest in the 1926–2012 period (Supplementary Table S4), we can speculate whether the strong correspondence of our approach with intensive monitoring is mostly valid under cold/limiting conditions and whether at least some inferences derived from intensive monitoring should only be extrapolated to past colder periods with caution. In other words, plant responses to future climate scenarios could have a higher level of uncertainty if the projections are based just on short-term, although intense, monitoring. Integration with long-term retrospective analyses could therefore help to improve our knowledge and produce a more reliable output.

## Conclusion

To analyze and thoroughly understand ecological processes we often need methods that integrate different spatial and temporal scales (Chave, 2013). Past studies on cambium dynamics have largely restricted their attention to the influence of concurrent climate conditions (Ogle et al., 2015). We are convinced that the application of wood anatomical traits over long time frames in a broad range of scientific contexts is worthwhile. Indeed, it can provide new possibilities for a better understanding of the mechanisms related to plant growth–environment interactions and better deciphers the information retained in tree-ring sequences, with a higher temporal resolution than ring-width analysis (Eilmann et al., 2009; Fonti et al., 2010; DeSoto et al., 2011; Olano et al., 2013).

We show that robust annually resolved wood anatomical measurements partitioned in intra-ring sectors, coupled with an adequate daily weather record, can provide information on cambium dynamics as accurate as that obtained by intensive monitoring. Dendroanatomical approaches do not allow the cambial activity and patterning of cell development to be followed over the growing season, but have the added value of providing a long-term perspective. This is a unique point of view dealing mostly with perennial and long-lived organisms and within a framework of long-term processes such as those related to climate change. There is certainly room for improvement: for example, additional anatomical or climate parameters such as cell wall thickness (Prendin et al., 2017) or precipitation could increase the quality of the information and the resulting inferences; tuning the length of the daily window could help to better relate growth processes to climate; the ring division in sectors, although it proved fairly effective (Carrer et al., 2016; Pellizzari et al., 2016; Castagneri et al., 2017a), can be considered a rigid and simplistic way to deal with the different and more plastic phenological phases. In the future, a ring partitioning that better considers individual- or species-specific physiological traits and tunes the time windows according to the yearly climate peculiarities, could provide better results.

Combining short-term intensive monitoring with long-term dendroanatomy offers new perspectives on the study of intra-annual growth processes. Reducing the uncertainties linked to plant phenological and growth responses to climate variability and change will likely foster reconstructions of past xylogenetic phases and improve the parameterization of future vegetation models by introducing a longer time frame.

## Author Contributions

MC designed the study and analyzed the data with input from all authors. MC, DC, GP, and AP provided the xylem anatomical data. MC, DC, and GvA wrote the article with input from all authors that finally read and approved the submitted version.

## Conflict of Interest Statement

The authors declare that the research was conducted in the absence of any commercial or financial relationships that could be construed as a potential conflict of interest.
