# Supplementary material for: Retrospective Analysis of Wood Anatomical Traits Reveals a Recent Extension in Tree Cambial Activity in Two High-Elevation Conifers
Source: Front Plant Sci. 2017 May 8;8:737. doi: 10.3389/fpls.2017.00737 (PMC5420594; doi:10.3389/fpls.2017.00737)
Supplement: Supplementary file 1 [file Table_1.docx]

**Table S1** Biometrical characteristics of sampled trees.

| **Species** | **Dbh (cm)** | **Height (m)** | **Age (years)** |
| --- | --- | --- | --- |
| *Picea abies* | 54.8 ± 13.1 | 20.0 ± 2.8 | 235 ± 45 |
| *Larix decidua* | 67.6 ± 5.9 | 22.7 ± 3.7 | 363 ± 82 |

Note: values are expressed as mean ± standard deviation.
