# Supplementary material for: Retrospective Analysis of Wood Anatomical Traits Reveals a Recent Extension in Tree Cambial Activity in Two High-Elevation Conifers
Source: Front Plant Sci. 2017 May 8;8:737. doi: 10.3389/fpls.2017.00737 (PMC5420594; doi:10.3389/fpls.2017.00737)
Supplement: Supplementary file 2 [file Table_2.docx]

**Table S2** Results of the Mann-Whitney U Test computed between the corresponding wood anatomical parameters in spruce and larch. All tests are significant at P < 0.001.

| **Decile** | **Rank Sum**  **spruce** | **Rank Sum**  **larch** | **U** | **Valid N**  **spruce** | **Valid N**  **larch** |
| --- | --- | --- | --- | --- | --- |
| **1st** | 244776.5 | 428603.5 | 15273.5 | 677 | 483 |
| **2 nd** | 237000.0 | 436380.0 | 7497.0 | 677 | 483 |
| **3 rd** | 237631.0 | 435749.0 | 8128.0 | 677 | 483 |
| **4 th** | 232142.5 | 441237.5 | 2639.5 | 677 | 483 |
| **5 th** | 232750.0 | 440630.0 | 3247.0 | 677 | 483 |
| **6 th** | 233406.5 | 439973.5 | 3903.5 | 677 | 483 |
| **7 th** | 242753.5 | 429466.5 | 13250.5 | 677 | 482 |
| **8 th** | 319459.5 | 352760.5 | 89956.5 | 677 | 482 |
| **9 th** | 468205.5 | 204014.5 | 87611.5 | 677 | 482 |
| **10 th** | 471611.5 | 190213.5 | 76210.5 | 673 | 477 |
| **CN** | 473278.5 | 146662.5 | 14307.5 | 599 | 514 |
