# Supplementary material for: Retrospective Analysis of Wood Anatomical Traits Reveals a Recent Extension in Tree Cambial Activity in Two High-Elevation Conifers
Source: Front Plant Sci. 2017 May 8;8:737. doi: 10.3389/fpls.2017.00737 (PMC5420594; doi:10.3389/fpls.2017.00737)
Supplement: Supplementary file 3 [file Table_3.docx]

**Table S3** Correlation values among ring width (TRW), cell number (CN) and decile chronologies (1926-2012) in both species. Non-significant values (P ≥ 0.05) are underlined.

| **Spruce** | | | | | | | | | | | |
| --- | --- | --- | --- | --- | --- | --- | --- | --- | --- | --- | --- |
| **Decile** | **1^st^** | **2^nd^** | **3^rd^** | **4 ^th^** | **5 ^th^** | **6 ^th^** | **7 ^th^** | **8 ^th^** | **9 ^th^** | **10^th^** | **TRW** |
| **1^st^** | 1.00 |  |  |  |  |  |  |  |  |  |  |
| **2 ^nd^** | 0.84 | 1.00 |  |  |  |  |  |  |  |  |  |
| **3 ^rd^** | 0.76 | 0.90 | 1.00 |  |  |  |  |  |  |  |  |
| **4 ^th^** | 0.62 | 0.77 | 0.89 | 1.00 |  |  |  |  |  |  |  |
| **5 ^th^** | 0.55 | 0.66 | 0.76 | 0.90 | 1.00 |  |  |  |  |  |  |
| **6 ^th^** | 0.53 | 0.58 | 0.66 | 0.77 | 0.88 | 1.00 |  |  |  |  |  |
| **7 ^th^** | 0.50 | 0.51 | 0.59 | 0.65 | 0.73 | 0.89 | 1.00 |  |  |  |  |
| **8 ^th^** | 0.38 | 0.40 | 0.44 | 0.52 | 0.59 | 0.71 | 0.89 | 1.00 |  |  |  |
| **9 ^th^** | 0.29 | 0.29 | 0.31 | 0.37 | 0.43 | 0.54 | 0.74 | 0.93 | 1.00 |  |  |
| **10 ^th^** | 0.28 | 0.30 | 0.33 | 0.35 | 0.38 | 0.45 | 0.62 | 0.81 | 0.92 | 1.00 |  |
| **TRW** | 0.46 | 0.40 | 0.32 | 0.20 | 0.22 | 0.32 | 0.38 | 0.37 | 0.37 | 0.33 | 1.00 |
| **CN** | 0.40 | 0.32 | 0.26 | 0.15 | 0.16 | 0.25 | 0.31 | 0.33 | 0.37 | 0.39 | 0.93 |
| **Larch** | | | | | | | | | | | |
| **Decile** | **1^st^** | **2^nd^** | **3^rd^** | **4 ^th^** | **5 ^th^** | **6 ^th^** | **7 ^th^** | **8 ^th^** | **9 ^th^** | **10^th^** | **TRW** |
| **1st** | 1.00 |  |  |  |  |  |  |  |  |  |  |
| **2 nd** | 0.71 | 1.00 |  |  |  |  |  |  |  |  |  |
| **3 rd** | 0.85 | 0.75 | 1.00 |  |  |  |  |  |  |  |  |
| **4 th** | 0.72 | 0.82 | 0.81 | 1.00 |  |  |  |  |  |  |  |
| **5 th** | 0.73 | 0.75 | 0.83 | 0.85 | 1.00 |  |  |  |  |  |  |
| **6 th** | 0.64 | 0.72 | 0.76 | 0.86 | 0.88 | 1.00 |  |  |  |  |  |
| **7 th** | 0.44 | 0.49 | 0.54 | 0.64 | 0.63 | 0.74 | 1.00 |  |  |  |  |
| **8 th** | 0.02 | 0.03 | 0.11 | 0.15 | 0.13 | 0.30 | 0.70 | 1.00 |  |  |  |
| **9 th** | -0.24 | -0.13 | -0.18 | -0.11 | -0.14 | 0.03 | 0.37 | 0.80 | 1.00 |  |  |
| **10 th** | -0.15 | 0.02 | -0.06 | 0.04 | 0.02 | 0.12 | 0.29 | 0.51 | 0.72 | 1.00 |  |
| **TRW** | 0.53 | 0.40 | 0.58 | 0.48 | 0.49 | 0.44 | 0.27 | 0.03 | -0.19 | -0.08 | 1.00 |
| **CN** | 0.60 | 0.51 | 0.62 | 0.54 | 0.58 | 0.48 | 0.23 | -0.22 | -0.50 | -0.24 | 0.73 |
