# Supplementary material for: Retrospective Analysis of Wood Anatomical Traits Reveals a Recent Extension in Tree Cambial Activity in Two High-Elevation Conifers
Source: Front Plant Sci. 2017 May 8;8:737. doi: 10.3389/fpls.2017.00737 (PMC5420594; doi:10.3389/fpls.2017.00737)
Supplement: Supplementary file 4 [file Table_4.docx]

**Table S4** The 30 coldest and warmest April-September years in the 1926-2012 period, as recorded at the Cortina d'Ampezzo meteorological station. Calendar years are listed in chronological order.

| **Coldest**  **Years** | **°C** |  | **Warmest**  **Years** | **°C** |
| --- | --- | --- | --- | --- |
| 1926 | 16.2 |  | 1928 | 17.7 |
| 1936 | 16.4 |  | 1929 | 18.3 |
| 1938 | 15.7 |  | 1932 | 17.4 |
| 1939 | 15.7 |  | 1934 | 18.2 |
| 1940 | 15.6 |  | 1935 | 17.4 |
| 1941 | 16.2 |  | 1942 | 17.3 |
| 1944 | 16.0 |  | 1943 | 17.6 |
| 1948 | 15.8 |  | 1945 | 17.5 |
| 1951 | 14.8 |  | 1947 | 18.2 |
| 1954 | 14.9 |  | 1952 | 17.7 |
| 1955 | 15.9 |  | 1961 | 18.4 |
| 1956 | 15.9 |  | 1963 | 17.3 |
| 1957 | 15.8 |  | 1983 | 17.5 |
| 1960 | 16.1 |  | 1985 | 17.5 |
| 1965 | 15.3 |  | 1990 | 17.5 |
| 1967 | 16.2 |  | 1992 | 17.5 |
| 1968 | 15.7 |  | 1994 | 17.6 |
| 1970 | 16.3 |  | 1998 | 17.9 |
| 1972 | 15.1 |  | 1999 | 18.0 |
| 1974 | 16.1 |  | 2000 | 18.6 |
| 1975 | 16.2 |  | 2001 | 17.6 |
| 1976 | 15.3 |  | 2002 | 17.4 |
| 1977 | 15.2 |  | 2003 | 20.2 |
| 1978 | 14.9 |  | 2005 | 17.3 |
| 1979 | 16.1 |  | 2006 | 17.6 |
| 1980 | 15.5 |  | 2007 | 18.1 |
| 1984 | 15.4 |  | 2009 | 18.4 |
| 1989 | 15.5 |  | 2010 | 17.3 |
| 1995 | 16.3 |  | 2011 | 19.1 |
| 1996 | 16.4 |  | 2012 | 18.1 |
| **Mean** | 15.8 |  | **Mean** | 17.9 |
