# Supplementary material for: Retrospective Analysis of Wood Anatomical Traits Reveals a Recent Extension in Tree Cambial Activity in Two High-Elevation Conifers
Source: Front Plant Sci. 2017 May 8;8:737. doi: 10.3389/fpls.2017.00737 (PMC5420594; doi:10.3389/fpls.2017.00737)

Figure S1: April-October daily temperature as recorded in 2001 (a) and 2008 (b) at the Cortina d'Ampezzo and Cinque Torri meteorological stations. Comparison (c) of the April-October daily temperature data recorded at the two stations for the period 1999-2012.

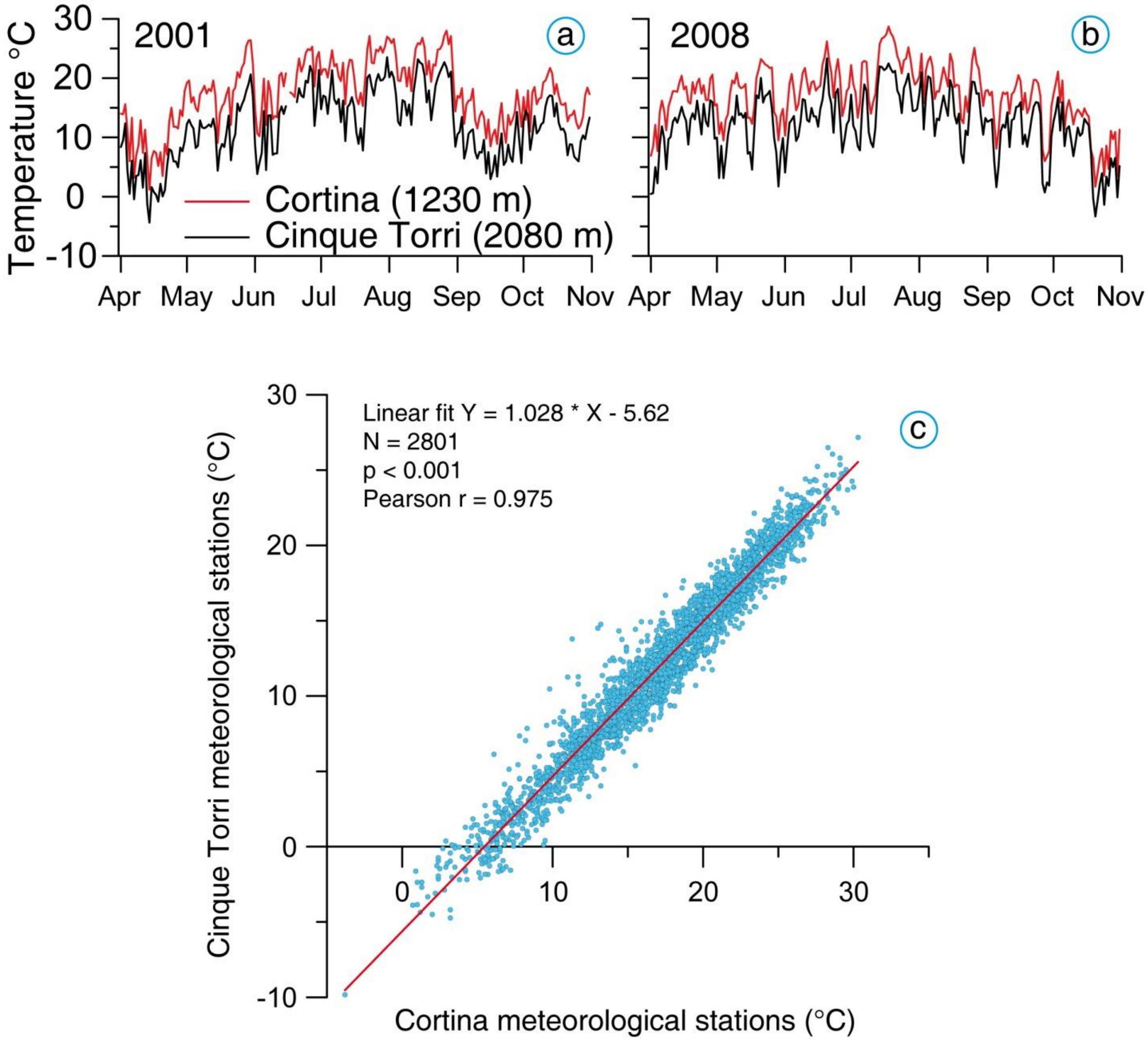

Supplement: Supplementary file 5 [file Image_1.pdf]
