# Supplementary material for: Retrospective Analysis of Wood Anatomical Traits Reveals a Recent Extension in Tree Cambial Activity in Two High-Elevation Conifers
Source: Front Plant Sci. 2017 May 8;8:737. doi: 10.3389/fpls.2017.00737 (PMC5420594; doi:10.3389/fpls.2017.00737)

Figure S2 Course of the mean monthly temperature for the 30 coldest and warmest April-September years listed in Table S4.

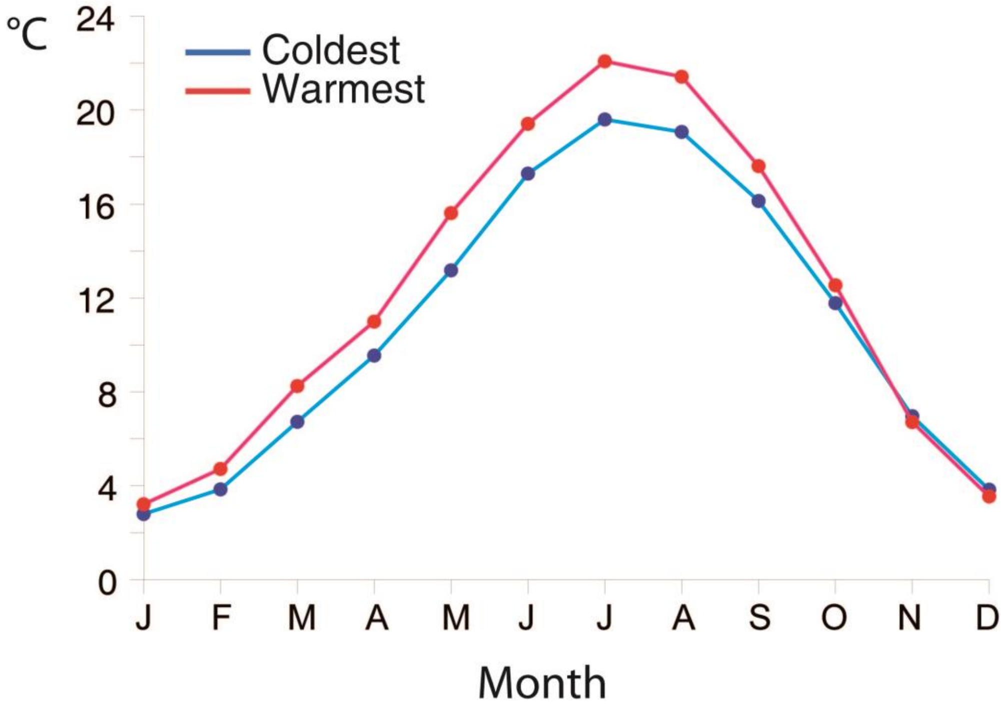

Supplement: Supplementary file 6 [file Image_2.pdf]
